# Comparative risk of QTc prolongation induced by second-generation antipsychotics in the real world: retrospective cohort study based on a hospital information system

**DOI:** 10.1192/bjo.2024.871

**Published:** 2025-03-10

**Authors:** Luyao He, Wenjuan Yu, Huiqing Song, Lujin Li, Yifeng Shen, Lei Zhang, Huafang Li

**Affiliations:** Department of Psychiatry, Shanghai Mental Health Center, Shanghai Jiao Tong University, School of Medicine, Shanghai, China; Shanghai Key Laboratory of Psychotic Disorders, Shanghai, China; Shanghai Null Hypothesis Information Technology Co. Ltd, Shanghai, China; Drug Clinical Research, Shanghai University of Traditional Chinese Medicine, Shanghai, China

**Keywords:** Atypical antipsychotic, cardiotoxicity, drug safety, electrocardiograph, medical records

## Abstract

**Background:**

Second-generation antipsychotics (SGAs) can cause corrected QT interval (QTc) prolongation as a side-effect. This may limit their clinical use and pose safety concerns for patients.

**Aims:**

To analyse the risk of QTc prolongation associated with eight second-generation antipsychotics and observe the timing characteristics of QTc prolongation events and subsequent changes in medication strategies.

**Methods:**

Using data from the hospital information system of a large mental health centre, this retrospective cohort study included 5130 patients (median follow-up: 141.2 days) treated between 2007 and 2019. A marginal structural Cox model was used to compare the hazard ratios for QTc prolongation associated with various SGAs.

**Results:**

The mean age of the cohort was 35.54 years (s.d. = 14.22), and 47.8% (*N* = 2454) were male. Ziprasidone, amisulpride and olanzapine were the only SGAs associated with QTc prolongation. Ziprasidone presented the highest risk (hazard ratio 1.72, 95% CI: 1.03–2.85, adjusted *P* = 0.03), followed by amisulpride (hazard ratio 1.56, 95% CI: 1.04–2.34, adjusted *P* = 0.03) and olanzapine (hazard ratio 1.40, 95% CI: 1.02–1.94, adjusted *P* = 0.04).

**Conclusion:**

Ziprasidone, amisulpride and olanzapine are associated with increased risk of QTc prolongation. Regular electrocardiogram monitoring is recommended when clinicians prescribe such drugs.

The corrected QT interval (QTc), measured from the onset of the Q wave to the end of the T wave on an electrocardiogram (ECG), is a parameter used in evaluation of drug safety with respect to adverse cardiac effects. A prolonged QTc interval may be associated with an increased risk of sudden cardiac death and can lead to torsades de pointes (TdP), a potentially fatal arrhythmia.^1,2^ The International Council for Harmonisation (ICH) recommends using QT/QTc to analyse adverse cardiac effects and issue guidelines for conducting a thorough QT or concentration QTc study before drug marketing to reduce cardiac adverse events.^3^

## Impact of antipsychotics on QTc prolongation

According to a report by Haddad et al, patients receiving antipsychotics have a two-fold risk of QTc prolongation compared with healthy individuals.^4^ Second-generation antipsychotics (SGAs) may cause QTc prolongation by blocking ion channels encoded by the human ether-à-go-go-related gene (hERG), which regulates cardiac action potential, a side-effect that restricts their clinical use and endangers patients.^5^ However, some SGAs have not undergone thorough QT study, making it difficult to estimate their risks and provide guidance for clinical practice.^6^

The association between QTc prolongation and SGAs has been a topic of extensive research. A meta-analysis of randomised controlled trials revealed that several SGAs may prolong QTc to varying extents. Compared with placebo, sertindole, ziprasidone and amisulpride significantly prolonged QTc by >20 ms, whereas olanzapine, risperidone and quetiapine marginally prolonged QTc by 5–15 ms.^7^ Therefore, SGAs may differ in their potential to induce QTc prolongation, and clinicians should consider this risk when prescribing antipsychotic drugs to patients with cardiac comorbidities or other risk factors. The meta-analysis had some limitations regarding follow-up periods and severity of mental illness in the study cohort. The studies included in the meta-analysis had follow-up periods of only 3–13 weeks; these were insufficient to enable complete evaluation of the cardiac risk due to QTc prolongation associated with SGAs. Moreover, the patients’ mental illnesses were generally less severe than those of a real-world population, which limited the applicability of the results.

Observational studies have also had weaknesses, such as not considering adequate variables, particularly laboratory reports, which may have compromised the validity and reliability of their results.^8–11^ Owing to the exclusion of these reports, it was impossible to adjust for factors such as electrolytes that influence QTc, potentially leading to biased results. As many factors influence QTc, inadequate control of confounding variables in observational studies may be a reason for the inconsistency between these studies’ findings and the results of the meta-analysis.^12^ Cohort studies that employ rigorous statistical methods and adequately adjust for confounding factors can establish causal relationships and provide more reliable evidence than cross-sectional studies. However, the large sample size required for cohort studies limits the number of available studies, especially when the incidence of drug-induced QTc prolongation is low (2–12%, depending on the drugs used).^13^

## Study rationale

Patients with schizophrenia often require SGAs for prolonged periods to achieve therapeutic benefits; thus, drug safety is particularly important for these patients. In this cohort study, we reviewed hospital records to compare the risk of QTc prolongation associated with different SGAs, determine the timing of QTc prolongation, and observe clinicians’ management of affected patients by tracking changes in in-patient prescriptions. These efforts aimed to provide robust evidence for clinical practice and offer recommendations for the management of high-risk populations.

## Methods

### Data source

This study was based on a database which leveraged in-patient electronic medical records and integrated personal identifying information of patients with schizophrenia from the hospital information system (HIS) of the Shanghai Mental Center Hospital. We extracted patient history descriptions, prescriptions, laboratory test results and ECG results from the medical record system and anonymised the data. Missing data were processed by searching the source files and performing multiple interpolations. Variables with >50% of missing values were excluded. This was a retrospective cohort study, and we obtained a waiver for informed consent from the ethics committee. Approval was obtained from the ethics committee of the Shanghai Mental Health Center (2019-16R), and the study was registered at clinicaltrials.gov (NCT04002258).

### Study population

All patients were treated in hospital at the Shanghai Mental Health Center between 1 February 2007 and 31 October 2019. A total of 19 370 in-patients with schizophrenia were identified in the HIS using *The International Statistical Classification of Diseases and Related Health Problems 10th Revision* (ICD-10). Patients prescribed aripiprazole, clozapine, quetiapine, olanzapine, ziprasidone, paliperidone, risperidone or amisulpride at admission were included in the study. Patients who met any of the following criteria were excluded: (a) evidence of long QT syndrome, acute cardiac failure, atrial fibrillation or atrial flutter; (b) lack of sex records; (c) absence of ECG records or only one ECG record during the study period; or (d) prolonged QTc on the first ECG examination at admission.

### Exposure and follow-up

Exposure was defined as continuous use of SGAs for 7 days from the initial in-patient prescription date. Exposure was compared eight times between the exposed and non-exposed groups, each for a different SGA. Patients underwent ECG examination on admission and monthly thereafter. If any abnormalities were detected, the frequency of the ECGs might have increased. Each ECG examination was considered a follow-up examination, and patients were followed up until discharge or the end of the study (31 October 2019).

### Outcome

The outcome was prolonged QTc. We defined QTc prolongation as QTc exceeding 450 ms in males and 470 ms in females, or increasing by >60 ms from baseline QTc, according to the ICH recommendation.^14^ QTc was corrected using the Bazett formula (QTc = QT (hazard ratio/60)^0.5^), a formula widely used in QTc prolongation studies and the default formula in electrocardiography.

### Covariates

Characteristics including demographic information, psychiatric medications, comorbidities, concomitant medications and supportive examinations were measured at admission and each ECG examination. For better adjustment of confounders, some variables were redefined. Hypokalaemia and hyperkalaemia were defined as serum potassium of <3.5 mmol/L and >5.5 mmol/L, respectively. The defined dose of antipsychotics was determined as olanzapine equivalents based on the conversion of the defined daily dose method.^15^ Patients’ concomitant medications were complex. To minimise bias owing to their impact on QTc, we categorised all non-study drugs, including other psychotropic medications, on the basis of their potential to affect QTc. The classification standard was obtained from the CredibleMeds website (https://www.crediblemeds.org; Supplementary Table 1, available at https://doi.org/10.1192/bjo.2024.871), which provides information on drug-induced TdP and QTc prolongation. Then, the numbers of patients receiving 0, 1 or ≥2 drugs that prolong QTc were determined.

### Statistical analysis

Measurement and count data were described as mean (s.d.) and number (%), respectively. To analyse the risk of SAG-induced QTc prolongation, we used the marginal structural Cox model, which constructs a counterfactual framework and pseudo-population through inverse probability weighting to simulate a comparison between the exposed and non-exposed groups.^16^ The pseudo-population was built using two weights, namely the treatment and deletion weights, which were estimated using the traditional Cox proportional hazards model. Baseline and time-dependent confounding variables were balanced after weighting. Eight comparisons were conducted between the exposed and non-exposed groups, each focusing on a specific target drug. For instance, when olanzapine was used as the target drug, the olanzapine group served as the exposed group. We analysed differences in the risk of QTc prolongation between the two groups. Consequently, the *P*-value was adjusted using the Benjamini–Hochberg procedure to reduce the probability of type I error (false positives).^17^ Statistical tests were two-sided, and *P* < 0.05 was considered to indicate statistical significance. All statistical analyses were performed using R software version 4.2.2.

## Results

### Demographics and clinical characteristics

**Fig. 1:**
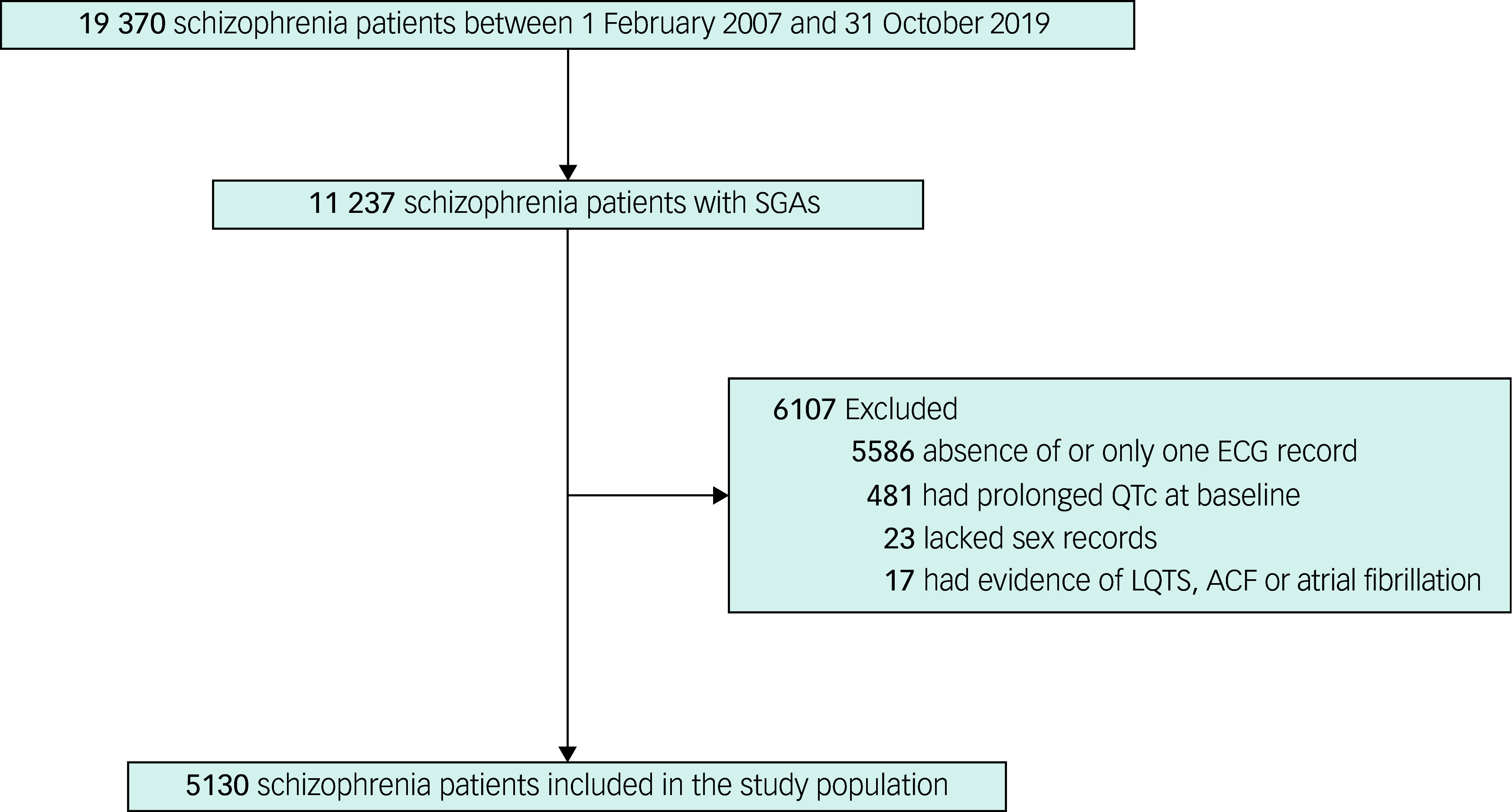
Flowchart of the study. ACF, acute cardiac failure; ECG, electrocardiogram; LQTS, long QT syndrome; QTc, corrected QT interval; SGAs, second-generation antipsychotics.

This study included 5130 patients who met the inclusion criteria (Fig. 1). The patients had a mean age of 35.54 years (s.d. = 14.22), and 47.8% were male (Table 1). The patients received treatment with various SGAs, with the following breakdown: olanzapine, *N* = 2238 (43.6%); quetiapine, *N* = 894 (17.4%); risperidone, *N* = 1700 (33.1%); clozapine, *N* = 837 (16.3%); aripiprazole, *N* = 809 (15.8%); paliperidone, *N* = 498 (9.7%); ziprasidone, *N* = 420 (8.2%); and amisulpride, *N* = 614 (12.0%). Patients were followed up for a mean duration of 141.2 days. During the study period, 260 patients experienced QTc prolongation. Supplementary Table 3 shows the numbers of patients who developed their first episode of QTc prolongation during the study period. QTc prolongation episodes were most frequent in the early stages; by the 12th week of the study period, 161 patients (61.9%) had experienced their first episode of QTc prolongation. The incidence of new episodes markedly decreased after the 20th week.

**Table 1:** Characteristics of the study population at baseline

Characteristic	All (*N* = 5130)
Age, mean (s.d.) years	35.54 (14.42)
Triglycerides, mean (s.d.) mmol/L	1.40 (1.08)
Cholesterol, mean (s.d.) mmol/L	4.60 (1.01)
Low-density lipoprotein, mean (s.d.) mmol/L	2.62 (0.86)
High-density lipoprotein, mean (s.d.) mmol/L	1.27 (0.33)
Oestradiol, mean (s.d.) pmol/L	167.33 (219.53)
Progesterone, mean (s.d.) nmol/L	4.25 (8.90)
Testosterone, mean, (s.d.) nmol/L	7.20 (7.64)
Follicle-stimulating hormone, mean (s.d.) U/L	13.20 (22.47)
Male, *n* (%)	2454 (47.8%)
Hypertension, *n* (%)	231 (5.3%)
Diabetes, *n* (%)	253 (5.8%)
Hypokalaemia, *n* (%)	517 (12.5%)
Patients treated with different numbers of QTc-prolonging drugs, *n* (%)	
0 drugs	3400 (66.3%)
1 drug	1300 (25.3%)
≥ 2 drugs	430 (8.4%)

### Risk of QTc prolongation associated with eight SGAs

**Fig. 2:**
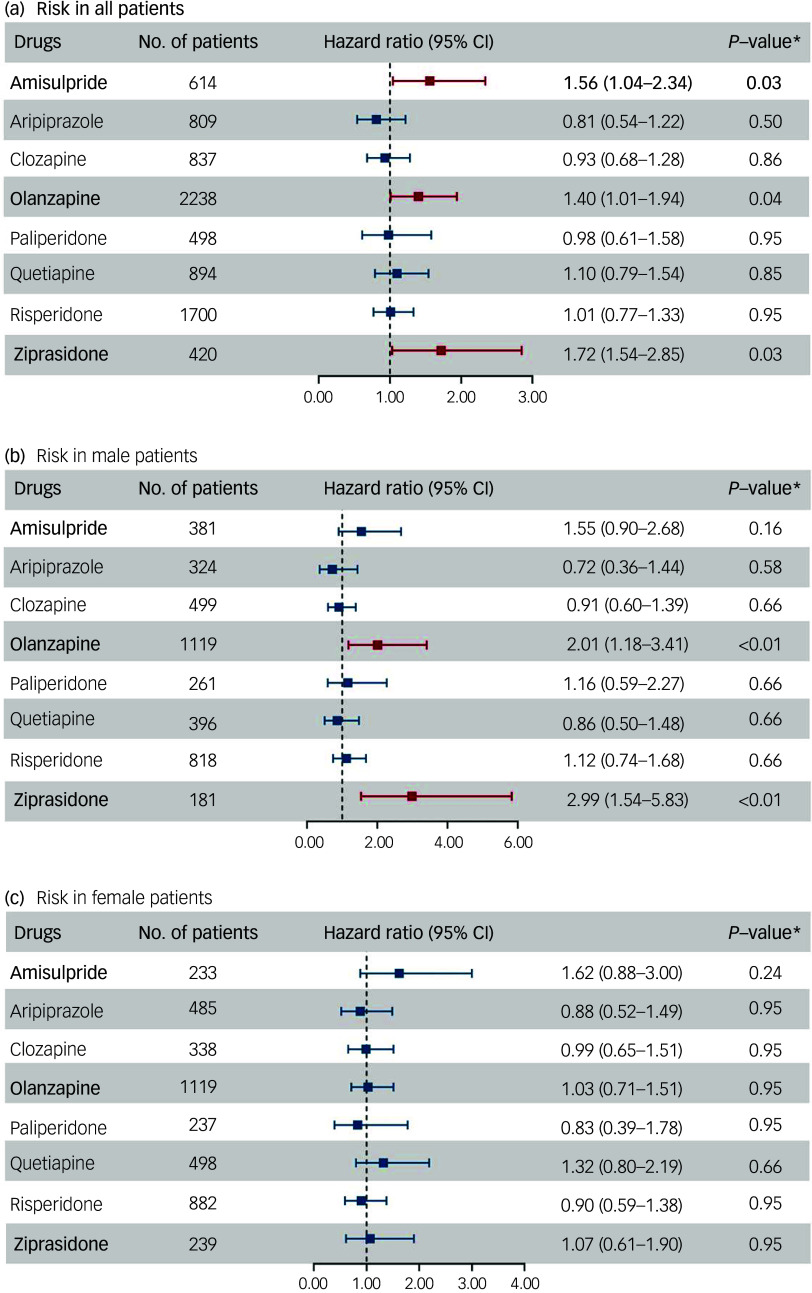
Risk of corrected QT (QTc) prolongation associated with specific second-generation antipsychotics. Asterisk indicates Benjamini–Hochberg-adjusted *P*-value.

Figure 2A presents the results of the marginal structural Cox model, which accounted for the confounding effects of both baseline and time-varying covariates (Supplementary Table 2) on the association between SGAs and QTc prolongation. Ziprasidone had a significant association with QTc prolongation (hazard ratio 1.72, 95% CI: 1.03–2.85, adjusted *P* = 0.03), whereas amisulpride had a lower risk (hazard ratio 1.56, 95% CI: 1.04–2.34, adjusted *P* = 0.03), similar to that of olanzapine (hazard ratio 1.40, 95% CI: 1.02–1.94, adjusted *P* = 0.04). No significant association was found between other SGAs (risperidone, clozapine, quetiapine, paliperidone, and aripiprazole) and QTc prolongation before or after *P*-value adjustment. In addition, we examined the effect of sex, and discovered that olanzapine (hazard ratio 2.01, 95% CI: 1.18–3.41, adjusted *P* < 0.01) and ziprasidone (hazard ratio 2.99, 95% CI: 1.54–5.83, adjusted *P* < 0.01) were significantly associated with the development of QTc prolongation in males but not in females (Fig. 2B and C).

### Description of treatment strategy after developing QTc prolongation

**Fig. 3:**
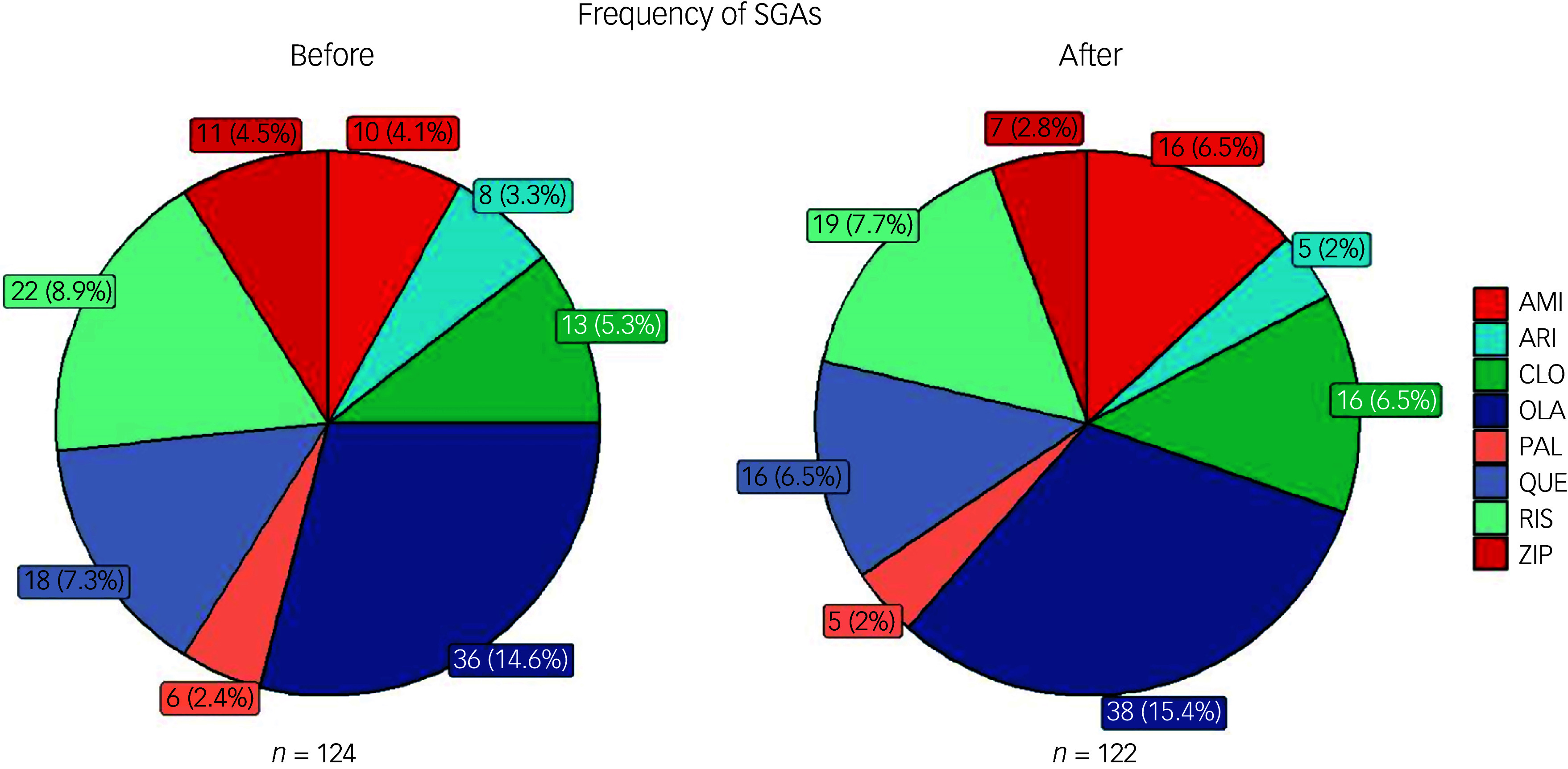
Frequencies of second-generation antipsychotic (SGA) prescriptions before and after the initial corrected QT (QTc) prolongation episode in 102 patients. AMI, amisulpride; ARI, aripiprazole; CLO, clozapine; OLA, olanzapine; PAL, paliperidone; QUE, quetiapine; RIS, risperidone; ZIP, ziprasidone. *N* is the total frequency of SGA prescriptions before or after the initial QTc prolongation episode in 102 patients.

Of the 260 patients who experienced QTc prolongation, 102 (39.2%) experienced more than one episode. Among them, 60 (58.8%) had their treatment strategy modified after the first episode by either reducing the dose or changing the medication. We evaluated changes in the frequency of SGAs before and after the first QTc prolongation event in the 102 patients (Figure 3). Before the first episode of QTc prolongation, olanzapine, ziprasidone and amisulpride were administered to 36 (14.6%), 11 (4.5%) and 10 patients (4.1%), respectively. After the first episode, the frequencies of olanzapine and amisulpride use increased to 38 (15.4%) and 16 patients (6.5%), respectively, whereas that of ziprasidone use decreased to seven patients (2.8%).

## Discussion

Using in-patient electronic medical records, we observed an association between QTc prolongation and use of ziprasidone, amisulpride and olanzapine in patients with schizophrenia. We also identified management challenges for patients with QTc prolongation by reviewing event history. Our findings provide real-world evidence of the safety of SGAs and their implications for clinical practice.

Our findings concurred with the results of a meta-analysis showing that amisulpride has a greater potential to prolong QTc than ziprasidone, and that the effect of olanzapine in this regard is weaker than that of risperidone.^7^ This discrepancy in results may be attributable to the different study methods used, particularly the definition of QTc prolongation. Although the risks associated with olanzapine and amisulpiride were significant, the lower bounds of their confidence intervals were close to 1. The limited sample size may explain the findings for amisulpride, although the reasons for its use are more complex. We adjusted for several covariates with potential mediating effects on QTc prolongation when constructing the marginal structural Cox model. Several studies have shown that lipid levels affect QTc, and olanzapine is known to frequently cause lipid abnormalities^18,19^; however, there is currently no direct evidence that lipid levels mediate olanzapine-induced QTc prolongation. Therefore, the adjustment may have led to underestimation of the risk associated with olanzapine administration. There is a paucity of real-world studies comparing the risks of QTc prolongation associated with different antipsychotics. Thus, our results require further confirmation in studies with larger sample sizes.^6^

Antipsychotics may induce QTc prolongation by blocking hERG channels, a mechanism suggested by patch-clamp studies of other drugs that have this effect. However, *in vitro* studies have shown that ziprasidone binds strongly to hERG channel receptors, with only sertindole binding more strongly, whereas olanzapine binds weakly to them.^20^ Therefore, ziprasidone may increase the risk of QTc prolongation by interfering with hERG channels, whereas olanzapine may exert this effect through alternative mechanisms. Therefore, the mechanism of antipsychotic-drug-induced QTc prolongation requires further investigation, and olanzapine may be a suitable candidate for study of this mechanism.

Clinicians were well aware of the risk of ziprasidone, as they often adjusted pharmacological strategies when patients treated with ziprasidone showed QTc prolongation, usually by stopping ziprasidone or switching it for another drug, consistent with guidelines.^1,21^ However, we found that olanzapine and amisulpride were often used as alternative drugs in cases of QTc prolongation. Olanzapine is a commonly used SGA worldwide with a lower discontinuation rate than other first-line SGAs in long-term studies.^22^ In China, olanzapine remains the second most commonly prescribed SGA according to a national survey.^23^ Olanzapine has consistently been used under the assumption of low risk, but there is still debate regarding its potential to cause QTc prolongation.^24,25^ Although no additional evidence suggests that switching to olanzapine or amisulpride leads to a further increase in the risk of QTc prolongation, we advise that these two drugs should be carefully selected as alternatives, with a balance between efficacy and safety in mind.

Furthermore, drug-induced QTc prolongation may involve sex-specific effects. An innovative study that used machine learning to generate synthetic data from cardiac myocyte models to simulate drug effects revealed sex-specific impacts on TdP, leading to the development of new sex-specific classification frameworks for TdP risk.^26^ The mechanism underlying these effects may involve an interplay among sex hormones, ion channels, drugs and other factors.^26,27^ Suzuki et al discovered sex differences in QTc across four SGAs (olanzapine, risperidone, aripiprazole and quetiapine), with women showing more variation than men.^28^ This suggests the presence of sex-specific effects in SGA-induced QTc prolongation. Our study confirmed this, with the only exception being women who received ziprasidone and olanzapine. This discrepancy could be attributed to the unequal sample sizes of the drugs examined. Alternatively, men may experience greater interactions with other risk variables.^29^ Thus, our results require further validation using larger and more balanced samples.

In this study, most patients experienced QTc prolongation for the first time within 12 weeks of follow-up. It has been reported that antipsychotic-naive first-episode in-patients with schizophrenia experience significant QTc prolongation from baseline after 2–4 weeks of antipsychotic use.^30^ We also observed that the frequency decreased and stabilised after 16–20 weeks. According to the American Heart Association guidelines on drug-induced arrhythmia, patients receiving potentially risky drugs are recommended to undergo ECG measurements every 3–6 months.^1^ We cannot eliminate the possibility that clinicians focus more on ECG monitoring and order more tests earlier in these cases. However, we recommend appropriately increasing the frequency of ECG monitoring within the first 16 weeks, and then adjusting it based on the actual situation.

This study had some limitations. Although we defined QTc for purposes of the study, no current consistent definition of QTc prolongation exists, and different studies have used different criteria according to their objectives. This is based on the ICH E14 document, American Heart Association guidelines and Chinese epidemiological findings,^1,5,14,31^ and we used a comprehensive approach, aiming to ensure inclusion of high-risk groups that might otherwise be overlooked. In addition, our study focused solely on single-drug analyses and did not investigate the effects of drug combinations. Therefore, we could not predict whether specific drug combinations were associated with an increased risk. For instance, when a patient is concurrently treated with olanzapine, quetiapine and sulpiride, it remains unclear whether the combination increases the risk of QTc prolongation. We excluded 5586 patients who had had only a single electrocardiogram, which may have introduced selection bias. However, our study design necessitated exclusion of patients with baseline QTc abnormalities, making this bias unavoidable. Finally, our study was a retrospective, non-randomised study, which implies that residual confounding factors could not be excluded, even after adjustment for age, sex and laboratory tests. Future studies should focus on investigating specific drug combinations and include larger sample sizes.

Using a retrospective cohort based on an HIS, we found that ziprasidone, amisulpride and olanzapine were associated with QTc prolongation, and that this risk was sex dependent, with higher risks in men receiving olanzapine and ziprasidone. Clinicians should consider the risks associated with these drugs when prescribing them, and regular ECG monitoring is essential. The monitoring frequency can be tailored to the long-term duration of medication use.

## Supporting information

He et al. supplementary material He et al. supplementary material

## Data Availability

The data generated and analysed during this study are available upon reasonable request from the corresponding author. Access to the data may be subject to restrictions to ensure confidentiality and compliance with relevant regulations.
