# Supplementary material for: Comparative risk of QTc prolongation induced by second-generation antipsychotics in the real world: retrospective cohort study based on a hospital information system
Source: BJPsych Open. 2025 Mar 10;11(2):e42. doi: 10.1192/bjo.2024.871 (PMC12001931; doi:10.1192/bjo.2024.871)
Supplement: He et al. supplementary material [file S2056472424008718sup001.docx]

**Supplemental Materials**

**Comparative the risk of QTc prolongation induced by second-generation antipsychotics in the real world: a retrospective cohort study based on Hospital Information System**

[Table S1. The list of drugs with known TdP risk and avoid in congenital Long QT 2](#_Toc23350)

[Table S2. List of Baseline and Time-Varying Covariates 5](#_Toc13791)

[Table S3. The number of patients who developed their first QTc prolongation over the study period 6](#_Toc13223)

[Figure S1. Distribution of stabilized weights at any point in the study period 7](#_Toc18485)

[Figure S2. The survival curves for the eight medications 8](#_Toc31566)

**Table S1. The list of drugs with known TdP risk and avoid in congenital Long QT**

| **Generic Name** | **Brand Names (Partial List)** | **Drug Class** | **Therapeutic Use** |
| --- | --- | --- | --- |
| Aclarubicin  *(Only on Non US Market)* | Aclacin, Aclacinomycine, Aclacinon, Aclaplastin, Jaclacin | Anti-cancer | Cancer |
| Amiodarone | Cordarone, Pacerone, Nexterone | Antiarrhythmic | Arrhythmia |
| Anagrelide | Agrylin, Xagrid | Phosphodiesterase 3 inhibitor | Thrombocythemia |
| Arsenic trioxide | Trisenox | Anti-cancer | Cancer (leukemia) |
| Astemizole *(Removed from US Market)* | Hismanal | Antihistamine | Allergic rhinitis |
| Azithromycin | Zithromax, Zmax | Antibiotic | Bacterial infection |
| Bepridil | Vascor | Antianginal | Angina Pectoris (heart pain) |
| Cesium Chloride | Energy Catalyst | Toxin | Alternative therapy cancer |
| Chloroquine | Aralen | Antimalarial | Malaria |
| Chlorpromazine | Thorazine, Largactil, Megaphen | Antipsychotic / Antiemetic | Nausea, Schizophrenia, many others |
| Chlorprothixene *(Only on Non US Market)* | Truxal | Antipsychotic | Schizophrenia |
| Cilostazol | Pletal | Phosphodiesterase 3 inhibitor | Intermittent claudication |
| Ciprofloxacin | Cipro, Cipro-XR, Neofloxin | Antibiotic | Bacterial infection |
| Cisapride *(Removed from US Market)* | Propulsid | GI stimulant | Increase GI motility |
| Citalopram | Celexa, Cipramil | Antidepressant, SSRI | Depression |
| Clarithromycin | Biaxin, Prevpac | Antibiotic | Bacterial infection |
| Cocaine | Cocaine | Local anesthetic | Anesthesia (topical) |
| Disopyramide | Norpace | Antiarrhythmic | Arrhythmia |
| Dofetilide | Tikosyn | Antiarrhythmic | Arrhythmia |
| Domperidone *(Only on Non US Market)* | Motilium, Motillium, Motinorm Costi, Nomit | Antiemetic | Nausea, vomiting |
| Donepezil | Aricept | Cholinesterase inhibitor | Dementia (Alzheimer's Disease) |
| Dronedarone | Multaq | Antiarrhythmic | Arrhythmia |
| Droperidol | Inapsine, Droleptan, Dridol, Xomolix | Antipsychotic / Antiemetic | Anesthesia (adjunct), nausea |
| Erythromycin | E.E.S., Robimycin, EMycin, Erymax, Ery-Tab, Eryc Ranbaxy, Erypar, Eryped, Erythrocin Stearate Filmtab, Erythrocot, E-Base, Erythroped, Ilosone, MY-E, Pediamycin, Abboticin, Abboticin-ES, Erycin, PCE Dispertab, Stiemycine, Acnasol, Tiloryth | Antibiotic | Bacterial infection, increase GI motility |
| Escitalopram | Cipralex, Lexapro, Nexito, Anxiset-E, Exodus, Esto, Seroplex, Elicea, Lexamil, Lexam, Entact, Losita, Reposil, Animaxen, Esitalo, Lexamil | Antidepressant, SSRI | Depression (major), anxiety disorders |
| Flecainide | Tambocor, Almarytm, Apocard, Ecrinal, Flécaine | Antiarrhythmic | Arrhythmia |
| Fluconazole | Diflucan, Trican | Antifungal | Fungal infection |
| Gatifloxacin *(Removed from US Market)* | Tequin | Antibiotic | Bacterial infection |
| Grepafloxacin *(Removed from US Market)* | Raxar | Antibiotic | Bacterial infection |
| Halofantrine *(Only on Non US Market)* | Halfan | Antimalarial | Malaria |
| Haloperidol | Haldol, Aloperidin, Bioperidolo, Brotopon, Dozic, Duraperidol, Einalon S, Eukystol, Halosten, Keselan, Linton, Peluces, Serenace, Serenase, Sigaperidol | Antipsychotic | Schizophrenia, agitation |
| Hydroquinidine (Dihydroquinidine) *(Only on Non US Market)* | Serecor | Antiarrhythmic | Arrhythmia |
| Hydroxychloroquine | Plaquenil, Quineprox | Antimalarial, Anti-inflammatory | Malaria, SLE, rheumatoid arthritis |
| Ibogaine *(Only on Non US Market)* |  | Psychedelic | Narcotic addiction, unproven |
| Ibutilide | Corvert | Antiarrhythmic | Arrhythmia |
| Levofloxacin | Levaquin, Tavanic | Antibiotic | Bacterial infection |
| Levomepromazine (Methotrimeprazine) *(Only on Non US Market)* | Nosinan, Nozinan, Levoprome | Antipsychotic | Schizophrenia |
| Levomethadyl acetate *(Removed from US Market)* | Orlaam | Opioid agonist | Narcotic dependence |
| Levosulpiride *(Only on Non US Market)* | Lesuride, Levazeo, Enliva | Antipsychotic | Schizophrenia |
| Meglumine antimoniate *(Only on Non US Market)* | Glucantime | Antiparasitic | Leishmaniasis |
| Mesoridazine *(Removed from US Market)* | Serentil | Antipsychotic | Schizophrenia |
| Methadone | Dolophine, Symoron, Amidone, Methadose, Physeptone, Heptadon | Opioid agonist | Narcotic dependence, pain |
| Moxifloxacin | Avelox, Avalox, Avelon | Antibiotic | Bacterial infection |
| Nifekalant *(Only on Non US Market)* | Shinbit | Antiarrhythmic | Arrhythmia |
| Ondansetron | Zofran, Anset, Ondemet, Zuplenz, Emetron, Ondavell, Emeset, Ondisolv, Setronax | Antiemetic | Nausea, vomiting |
| Oxaliplatin | Oxaliplatin | Anti-cancer | Cancer |
| Papaverine HCl *(Intra-coronary)* |  | Vasodilator, Coronary | Diagnostic adjunct |
| Pentamidine | Pentam | Antifungal | Fungal infection (Pneumocystis pneumonia) |
| Pimozide | Orap | Antipsychotic | Tourette's Disorder |
| Probucol *(Removed from US Market)* | Lorelco | Antilipemic | Hypercholesterolemia |
| Procainamide | Pronestyl, Procan | Antiarrhythmic | Arrhythmia |
| Propofol | Diprivan, Propoven | Anesthetic, general | Anesthesia |
| Quinidine | Quinaglute, Duraquin, Quinact, Quinidex, Cin-Quin, Quinora | Antiarrhythmic | Arrhythmia |
| Roxithromycin *(Only on Non US Market)* | Rulide, Xthrocin, Roxl-150, Roxo, Surlid, Rulide, Biaxsig, Roxar, Roximycinv, Roxomycin, Rulid, Tirabicin, Coroxin | Antibiotic | Bacterial infection |
| Sertindole *(Only on Non US Market)* | Serdolect, Serlect | Antipsychotic, atypical | Schizophrenia, anxiety |
| Sevoflurane | Ultane, Sojourn | Anesthetic, general | Anesthesia |
| Sotalol | Betapace, Sotalex, Sotacor | Antiarrhythmic | Arrhythmia |
| Sparfloxacin *(Removed from US Market)* | Zagam | Antibiotic | Bacterial infection |
| Sulpiride *(Only on Non US Market)* | Dogmatil, Dolmatil, Eglonyl, Espiride, Modal, Sulpor | Antipsychotic, atypical | Schizophrenia |
| Sultopride *(Only on Non US Market)* | Barnetil, Barnotil, Topral | Antipsychotic, atypical | Schizophrenia |
| Terfenadine *(Removed from US Market)* | Seldane | Antihistamine | Allergic rhinitis |
| Terlipressin *(Only on Non US Market)* | Teripress, Glypressin, Terlipin, Remestyp, Tresil, Teriss | Vasoconstrictor | Septic shock |
| Terodiline *(Only on Non US Market)* | Micturin, Mictrol | Muscle relaxant | Bladder spasm |
| Thioridazine | Mellaril, Novoridazine, Thioril | Antipsychotic | Schizophrenia |
| Vandetanib | Caprelsa | Anti-cancer | Cancer (thyroid) |

**Table S2. List of Baseline and Time-Varying Covariates**

| ***Baseline Covariates*** |  |
| --- | --- |
| Age | Male |
| Triglyceride | Hypertension |
| Cholesterol | Diabetes |
| Low density lipoprotein | Hypokalemia |
| High density lipoprotein | Drugs that cause prolonged QTc |
| Estradiol | 0 |
| Progesterone | 1 |
| Testosterone | ≥ 2 |
| Follicle-stimulating hormone |  |
| ***Time-Varying Covariates*** |  |
| Age | Hypertension |
| Triglyceride | Diabetes |
| Cholesterol | Hypokalemia |
| Low density lipoprotein | Drugs that cause prolonged QTc |
| High density lipoprotein | 0 |
| Estradiol | 1 |
| Progesterone | ≥ 2 |
| Testosterone |  |
| Follicle-stimulating hormone |  |

**Table S3. The number of patients who developed their first QTc prolongation over the study period**

| Study period, weeks | Numbers of Patients developed QTc prolongation, n(%) | Cumulative numbers of patients developed QTc prolongation, n(%) |
| --- | --- | --- |
| ≦4 | 72(27.7%) | 72(27.7%) |
| 4-8 | 57(21.9%) | 129(49.6%) |
| 8-12 | 32(12.3%) | 161(61.9%) |
| 12-16 | 16(6.2%) | 177(68.1%) |
| 16-20 | 10(3.8%) | 187(71.9%) |
| 20-24 | 4(1.5%) | 191(73.5%) |
| 24-28 | 3(1.2%) | 194(74.6%) |
| 28-32 | 2(0.8%) | 196(75.4%) |
| 32-36 | 3(1.2%) | 199(76.5%) |
| 36-40 | 4(1.5%) | 203(78.1%) |
| 40-44 | 1(0.4%) | 204(78.5%) |
| 44-48 | 6(2.3%) | 210(80.8%) |
| 48-52 | 3(1.2%) | 213(81.9%) |
| >52 | 47(18.1%) | 260(100.0%) |


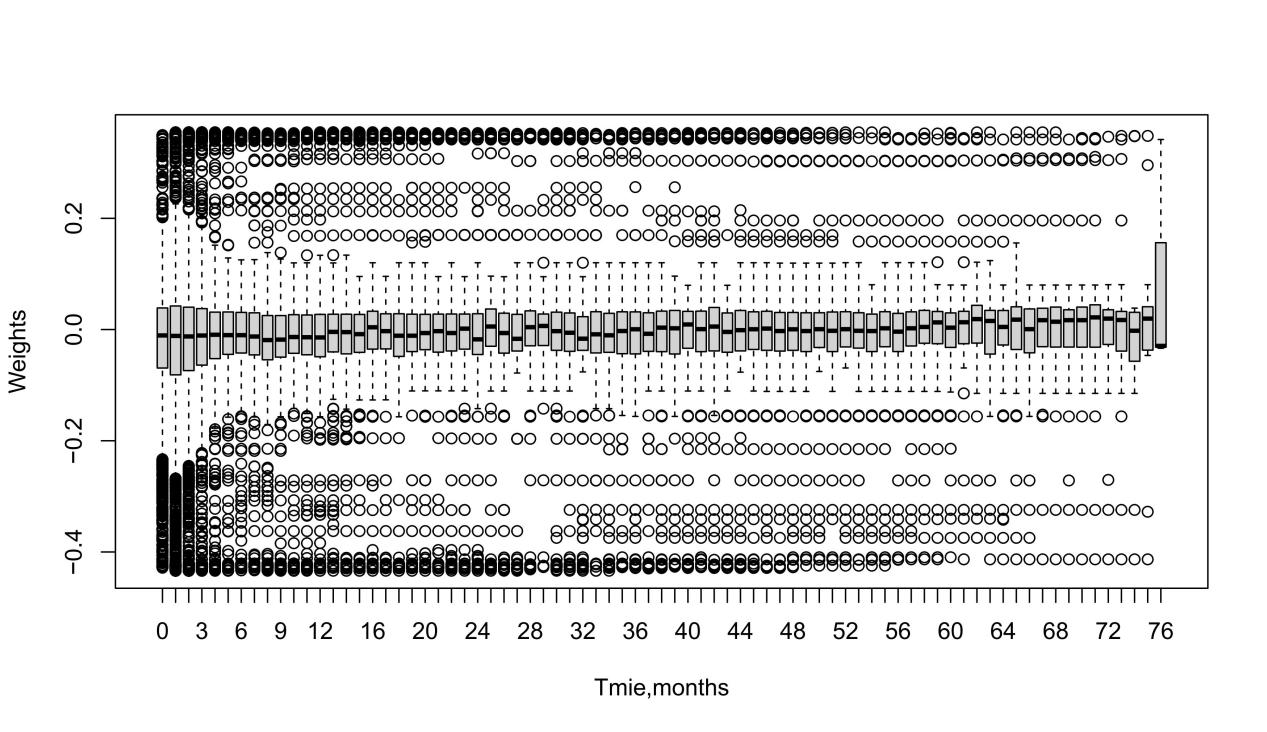


**Figure S1. Distribution of stabilized weights at any point in the study period.** The box displays the summary statistics of the data, including the mean (+), median (horizontal line inside the box), interquartile range (length of the box), and outliers (circles). The whiskers extend to the minimum and maximum values.


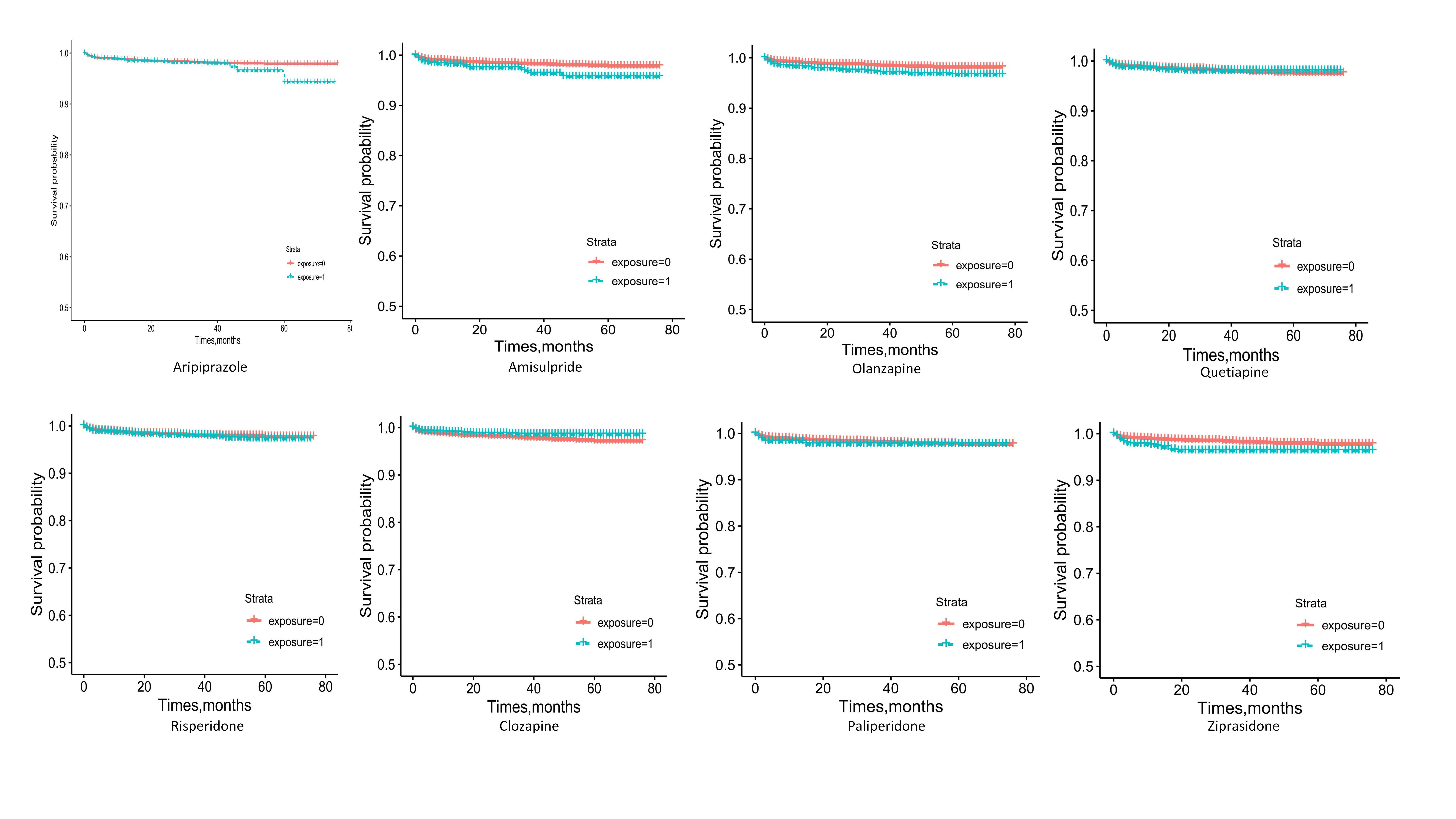


**Figure S2. The survival curves for the eight medications.** The survival curves illustrate the time to reach prolonged QTc for each of the eight antipsychotic medications analyzed in the study. Each curve reflects the proportion of patients remaining free of prolonged QTc over time, providing insights into the duration and risk associated with each medication.
